# Multi-layered ZIF-coated cells for the release of bioactive molecules in hostile environments†

**DOI:** 10.1039/d2cc03072a

**Published:** 2022-08-01

**Authors:** Lei Gan, Miriam de J. Velásquez-Hernández, Anita Emmerstorfer-Augustin, Peter Wied, Heimo Wolinski, Simone Dal Zilio, Marcello Solomon, Weibin Liang, Christian Doonan, Paolo Falcaro

**Affiliations:** Institute of Physical and Theoretical Chemistry, Graz University of Technology, Stremayrgasse 9 Graz 8010 Austria paolo.falcaro@tugraz.at; Institute of Molecular Biotechnology, Graz University of Technology, NAWI Graz, BioTechMed-Graz,, Petergasse 14 Graz 8010 Austria; Institute of Molecular Biosciences, BioTechMed-Graz, University of Graz Graz Austria; Istituto Officina dei Materiali CNR, Basovizza, Edificio MM-SS Trieste Italy; School of Physical Sciences, Faculty of Sciences, University of Adelaide South Australia 5005 Australia christian.doonan@adelaide.edu.au

## Abstract

Metal–organic framework (MOF) coatings on cells enhance viability in cytotoxic environments. Here, we show how protective multi-layered MOF bio-composite shells on a model cell system (yeast) enhance the proliferation of living cells exposed to hostile protease-rich environments *via* the dissolution of the shells and release of a protease inhibitor (antitrypsin).

## Abstract 2

Living cells are coated with a multi-layered MOF biocomposite shell: the shell dissolution releases a biotherapeutic (antitrypsin) that provides artificial adaptability to protease-rich environments.

## Graphical abstract

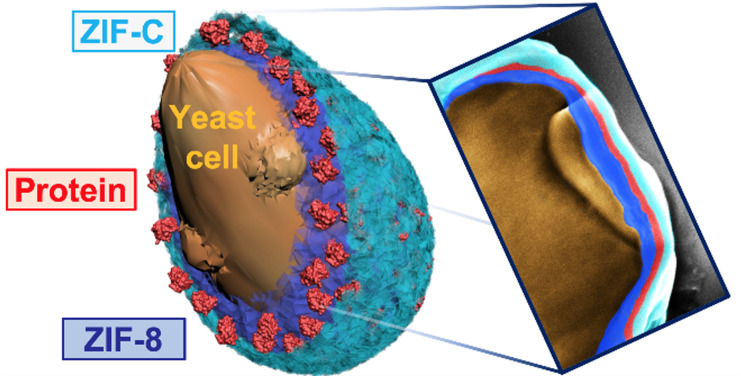

Molecular biology has demonstrated the importance of research on living cells and microorganisms for the advancement of biotechnological and biomedical applications.^1–3^ For example, *Moorella thermoacetica* bacterium was used for the reduction of CO_2_ to acetic acid,^4^ while stem cells have been applied to regenerative therapies, tissue engineering, and diagnosis.^5^ For these applications, cells are removed from their native environments and exposed to stressors (*e.g.*, high temperature, enzymatic degradation).^6^ The relocation of cells in non-native environments often leads to loss of cell viability and limits the bioactivity of the system.^7–10^ To enhance the resistance of cells exposed to hostile conditions, methods for encapsulating cells within abiotic exoskeletons are being studied.^7,9,11^ To this end, metal–organic frameworks (MOFs), a class of materials synthesized from metal ions interconnected by multidentate organic linkers,^12^ have been used to prepare cytoprotective coatings on living cells.^13^ Zeolitic imidazolate framework-8 (ZIF-8)^14^—a MOF composed of tetrahedral Zn^2+^ ions linked by 2-methylimidazolate (mIM)—was successfully used for the fabrication of abiotic shells.^15^ ZIF-8, in its porous form, has a crystalline lattice with sodalite (**sod**) topology.^14^ However, by changing Zn^2+^ : HmIM, different crystalline phases (*e.g.*, amorphous, diamondoid) with distinct mass transfer properties can be synthesized.^16–18^ ZIF-8 can be synthesized in water or buffer solutions^13^ and is known to self-assemble on naturally occurring bioentites, from proteins to bacteria.^19,20^ An additional feature of this chemistry is that the protective ZIF-8 coating can be simply removed from the cell by decreasing the pH,^21^ adding chelating agents (*e.g.*, ethylenediaminetetraacetic acid, EDTA),^22^ or exposing ZIF-8 to some buffer solutions (*e.g.*, phosphate-buffered saline, PBS).^23–25^ We have previously demonstrated the successful encapsulation of *Saccharomyces cerevisiae* (yeast cells) within **sod** ZIF-8 using a *one-pot* approach.^26^ In a few minutes, a *ca.* 100 nm thick ZIF-8 shell assembled on the yeast cell surface. This porous coating formed a cytoprotective barrier with perm-selective properties: *i.e.* it was permeable to nutrients (glucose) but not to larger cytotoxic molecules (*e.g.* lyticase).^26^ Thus, the yeast cell metabolic activity was maintained within the rigid abiotic shell even in the presence of cytotoxic molecules. Subsequent to the removal of the exoskeleton, the cells retained their biological functionality including reproduction.^26^ This ZIF-8-based coating strategy has been extended to viruses,^27^ bacteria^26^ and mammalian cells.^28^ Further research showed that yeast cells could be coated with a film of enzymes (β-galactosidase, β-gal) prior to the assembly of a protective ZIF-8 coating.^29^ The immobilized β-gal conferred biocatalytic properties to the exoskeleton by processing lactose into glucose (a cell nutrient). This study revealed that engineering abiotic shells with co-immobilized enzymes can enhance the viability of the encaged cells. To date, all the research on protective ZIF coatings has been focused on the shielding properties of ZIF-based exoskeletons as by using this cell@ZIF approach in an environment with overexpressed protease (*e.g.* inflammatory condition^30^ and some tumor environment^31^), cell death can be prevented. However, if this environment is the final destination of the cell, when the MOF shell is dissolved, exposure of the cell membrane to protease will cause cell death.^32^ To avoid degradation of the cell membrane subsequent to the removal of the ZIF-8 shell, one strategy could be to co-encapsulate a protease inhibitor that would be released during the MOF shell dissolution process. This would inactivate the cytotoxic enzyme in the surrounding environment and enable rapid cell proliferation.

In general, the potential of ZIF coatings to protect cells from environmental stressors and enhance cell thriving is still an undeveloped area of research. By showing that a biocomposite MOF shell could transform an environment from cytotoxic to biocompatible would further progress MOF materials for biotechnology and biomedicine. Here we demonstrate this proof-of-concept by using yeast as a model cell system, trypsin as a model protease enzyme, and Alpha-1-antitrypsin (AAT), a protein therapeutic, as a protease inhibitor.

Inspired by the affinity of proteins for ZIFs^33^ and the ability of protein-functionalized surfaces to trigger the ZIF formation^29^ we developed a multilayer approach to control the AAT encapsulation within two ZIF layers. Firstly, a **sod** ZIF-8 layer is grown on yeast cells (Fig. 1a and b), followed by adsorption of AAT on the yeast@ZIF-8 biocomposite (Fig. 1c). Then a second ZIF shell is grown to cover the immobilized AAT (Fig. 1d). We note that, depending on synthesis conditions, either a phase pure ZIF-8 or ZIF-C (*i.e.* Zn_2_(mIM)_2_(CO_3_))^34^ shell can be deposited. ZIF-C was unexpected as it has not been reported to form a cell coating. This non-porous framework, which includes CO_3_^2−^ from atmospheric CO_2_, was observed as a product of biomimetic mineralization from ZIF-8 precursors and proteins.^17^ Compared to ZIF-8, ZIF-C shows different release kinetics of encapsulated molecules.^17^ Thus, this multilayered approach enables: (1) control over the spatial distribution of the AAT in the MOF exoskeleton, (2) the release profile of AAT to be modified *via* the selection of the ZIF phase for the outer layer (ZIF-8 – slow release, or ZIF-C – fast release) and, (3) artificial adaptability to protease-rich environments (Fig. 1e–i).

**Fig. 1:**
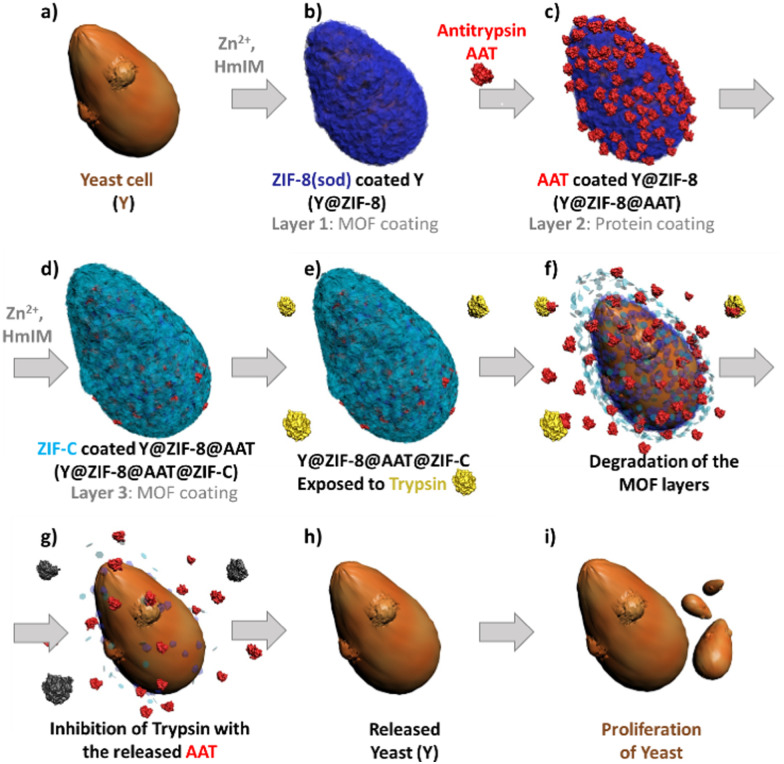
Schematic diagram of yeast (a) coated with a multistep approach: ZIF shell (b), protein film (c), second ZIF shell (d) and cell proliferation under released of the yeast in presence of trypsin (e–i).

*Saccharomyces cerevisiae* (yeast cells; Y) was selected as a model organism as it is robust, non-pathogenic, easy to cultivate and divides similarly to human cells.^35^ The first protective ZIF-8 shell was synthesized by adding Y to an aqueous solution with the MOF precursors (Zn^2+^ : HmIM = 1 : 16, ESI†). When compared to previously reported *one-pot* protocols,^26^ the current higher ligand to metal ratio affords the rapid crystallization of Zn(mIM)_2_ into pure **sod** ZIF-8 as confirmed by the powder X-ray diffraction (PXRD) pattern (Fig. S1, ESI†) of the washed samples. Scanning electron microscopy (SEM) analyses reveal the formation of a homogenous ZIF-8 coating with an average shell thickness of *ca.* 60 ± 20 nm (Fig. 2 and Fig. S2 and S3, ESI†). The analysis of the dried Y@ZIF-8 with Fourier-transform infrared (FTIR) spectroscopy shows characteristic modes of the Zn–N bond of ZIF-8 (*e.g.* 421 cm^−1^, Fig. S4, ESI†). Next, Bovine Serum Albumin (BSA) was selected as an inexpensive model protein for the optimization of a protein layer on the ZIF-8 exoskeleton. By soaking 9 mg of Y@ZIF-8 in an aqueous solution of BSA, 0.05 mg of the protein were adsorbed on the Y@ZIF-8 surface (ESI†). Finally, Y@ZIF-8@BSA was exposed to the ZIF-8 precursors. Using a Zn^2+^ : HmIM ratio of 1 : 32, self-assembled shells of pure **sod** ZIF-8 (Fig. 2a and Table S1, ESI†) were achieved whereas a Zn^2+^ : HmIM = 1 : 4 ratio yielded a crystalline shell of pure ZIF-C (Fig. 2a and Table S1, see ESI† for full experimental procedure). A 100% adsorption efficiency (AE) of BSA was determined for both bio-composites (Fig. S5–S7, Table S2 and S3, ESI†). The morphology of Y@ZIF-8@BSA@ZIF-8 and Y@ZIF-8@BSA@ZIF-C were analyzed by SEM. Close inspection of the images clearly shows the formation of continuous ZIF coatings in both samples (SEM, Fig. 2c and d). An average shell thickness, determined from the cross-section (focused ion beam, FIB, ESI†), of the ZIF shells was *ca.* 102 nm and *ca.* 95 nm, for Y@ZIF-8@BSA@ZIF-8 and Y@ZIF-8@BSA@ZIF-C, respectively (Fig. 2c, 2d and Fig. S8, S9, ESI†). To ascertain the effect of the different crystalline phases on the outer ZIF-shells on the protein secretion, we measured the release profile of BSA upon the digestion of ZIF in an aqueous solution of EDTA (fast trigger release) and phosphate buffer (PB, 20 mM, pH 6.5, simulating acidic conditions associated with cancerous cells^36^ and inflammation^37^). Release tests triggered by EDTA show a 100% BSA release in *ca.* 15 min for Y@ZIF-8@BSA@ZIF-C, and *ca.* 30 min for Y@ZIF-8@BSA@ZIF-8 (Fig. S10, ESI†). The release profiles were then determined in PB (Fig. S11, ESI†). The profile measured from Y@ZIF-8@BSA@ZIF-C shows an 80% release of BSA in *ca.* 2 h, while for Y@ZIF-8@BSA@ZIF-8, 80% release was observed in *ca.* 15 h. In both media the release from ZIF-C was always faster than that from ZIF-8. These results indicate that, for the same digestion environment, the release kinetics of the immobilized protein can be tuned by engineering the crystalline phase of the outer ZIF shell.

**Fig. 2:**
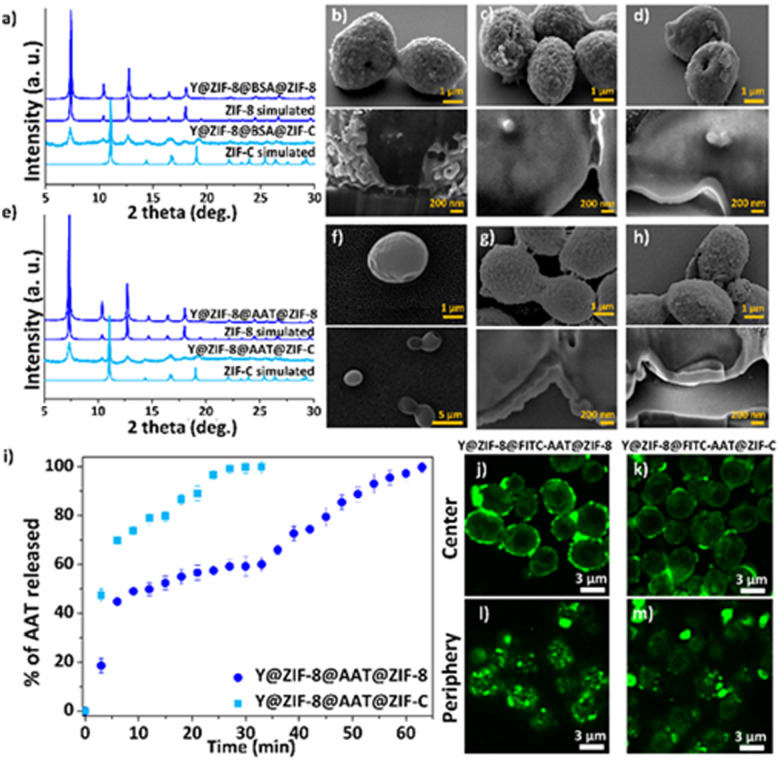
Powder X-ray diffraction (PXRD) of Y@ZIF-8@BSA@ZIF (ZIF = ZIF-8, ZIF-C) (a). SEM images and cross-section analysis of Y@ZIF-8 (b), Y@ZIF-8@BSA@ZIF-8 (c), Y@ZIF-8@BSA@ZIF-C (d). PXRD of Y@ZIF-8@AAT@ZIF (ZIF = ZIF-8, ZIF-C) (e). SEM images and cross-section analysis of Y (f), Y@ZIF-8@AAT@ZIF-8 (g), Y@ZIF-8@AAT@ZIF-C (h). Kinetic release profile of AAT released from Y@ZIF-8@AAT@ZIF (ZIF = ZIF-8, ZIF-C) upon exposing to EDTA (i). Single confocal optical sections (deconvolution data) taken from the centre of cells (j and k) and from the periphery (l and m).

Next, following the same protocols established with BSA, the fabrication of the multilayered system was tested with the clinical biotherapeutic AAT, and Y@ZIF-8@AAT@ZIF-8 and Y@ZIF-8@AAT@ZIF-C were prepared. The PXRD of Y@ZIF-8@AAT@ZIF-8 was analogous to pure ZIF-8 **sod** while for Y@ZIF-8@AAT@ZIF-C the pattern showed peaks attributed to both **sod** and ZIF-C (Fig. 2e). FTIR spectra of the biocomposites show modes attributed to the Zn–N bonds of the ZIF networks and the amide bonds from the proteins, 421 cm^−1^ and 1635 cm^−1^, respectively. Additionally, for the ZIF-C biocomposite, the 700–850 and 1300–1400 cm^−1^ bands were assigned to weak bending and asymmetric stretching of CO_3_^2−^ modes (Fig. S12, ESI†). The AAT adsorption efficiencies for Y@ZIF-8@AAT@ZIF-8 and Y@ZIF-8@AAT@ZIF-C were 100% (Fig. S7, Fig. S13–16, Table S4 and S5, ESI†). The morphology of the bio-composites was examined by SEM. Close inspection of the images revealed ellipsoidal monomodal particle distributions, suggesting that Y@ZIF-8@AAT particles act as seeds for the formation of the second ZIF-8 and ZIF-C layers. Furthermore, both ZIF-8 and ZIF-C coatings are continuous and possess a similar morphology to the BSA counterparts (rounded particle-like for ZIF-8 and plate-like for ZIF-C) (Fig. 2f–h). The average shell thicknesses obtained from Y@ZIF-8@AAT@ZIF-8 and Y@ZIF-8@AAT@ZIF-C are *ca.* 270 nm and *ca.* 125 nm, respectively (Fig. S17 and S18, ESI†). It is worth noting that the multilayer strategy inhibits the crystallization of independent AAT@ZIF-8 particles, which is not the case when yeast and AAT are simultaneously present during the ZIF formation (Fig. S19, ESI†).

The release profiles obtained from Y@ZIF-8@AAT@ZIF-8 and Y@ZIF-8@AAT@ZIF-C in EDTA and PB reveal trends similar to their BSA analogues (Fig. 2i and Fig. S20, ESI†). For example, in the digestion by EDTA, a 100% release of AAT is measured in 30 min for Y@ZIF-8@AAT@ZIF-C, while the full release takes 1 h for Y@ZIF-8@AAT@ZIF-8. For PB solution, a 50% release of AAT is measured in *ca.* 2 h for ZIF-C and again longer for ZIF-8 (*i.e. ca.* 18 h, ESI†). Confocal laser scanning microscopy (CLSM) was employed to assess the homogeneity and localization of the protein layer immobilized between the two ZIF layers. For this analysis, we used BSA and AAT tagged with fluorescein isothiocyanate (FITC-BSA, FITC-AAT) to synthesize the biocomposites. Fig. 2j and k show the center of Y@ZIF-8@FITC-AAT@ZIF-8 and Y@ZIF-8@FITC-AAT@ZIF-C, respectively. Fig. 2l and m show the periphery of Y@ZIF-8@FITC-AAT@ZIF-8 and Y@ZIF-8@FITC-AAT@ZIF-C composites (Videos S1, S2 and Fig. S21, ESI† for FITC-BSA samples, Fig. S22, ESI† for controls). In summary, the CLSM images confirm the successful immobilization of protein between the two ZIF layers on the yeast cells.

To evaluate whether AAT retains its protease inhibitor function after release from the ZIF shells, Y@ZIF-8@AAT@ZIF-8 and Y@ZIF-8@AAT@ZIF-C were digested in EDTA and PB, then the supernatant from the composite solutions were exposed to a trypsin solution. After 30 min storage at RT, the protease activity of trypsin was analyzed using a Trypsin Activity Assay (Fig. S23 and S24, ESI†). In PB, where the release is slower (Fig. S23 and S24, ESI†), we monitored the effect of AAT release from Y@ZIF-8@AAT@ZIF-8 on trypsin. Over the screened period, trypsin became increasingly inactivated by the release of AAT. For a 100% AAT release, trypsin become completely inhibited.

Finally, we ascertained the bio-protection functionality of AAT released from the ZIF coating on cells in a protease-rich environment. Y@ZIF-8@AAT@ZIF-8 and Y@ZIF-8@AAT@ZIF-C were directly exposed for 4 h to a trypsin solution (0.25 mg mL^−1^) with EDTA. The released yeast cells were washed with water, diluted, and resuspended in yeast growth medium (Yeast-extract-Peptone-Dextrose; YPD, ESI†).^38^ Cell proliferation was monitored by optical density measurements at 600 nm (OD_600_, ESI†).^38^ As control experiments, uncoated cells and the composites obtained with non-active protein (BSA) were exposed to trypsin under the same conditions used for AAT analogs. The OD_600_ measurements reveal that uncoated cells exposed to trypsin exhibit a longer lag phase (*ca.* 13 h) than the non-exposed cells (*ca.* 6 h, Fig. 3). The time difference (Δ*t* = 7 h) to initiate the exponential growth of cells demonstrates the detrimental effect of trypsin on uncoated cells. When comparing the Y@ZIF-8@BSA@ZIF-C and Y@ZIF-8@AAT@ZIF-C systems exposed to trypsin, a Δ*t* = 9 h was measured. Similarly, a Δ*t* = 8 h was measured when evaluating the time difference in the lag phases of Y@ZIF-8@BSA@ZIF-8 and Y@ZIF-8@AAT@ZIF-8. Collectively, these data demonstrate that, in a trypsin-rich environment, yeast cells reproduce faster when AAT is released by ZIF coatings.

**Fig. 3:**
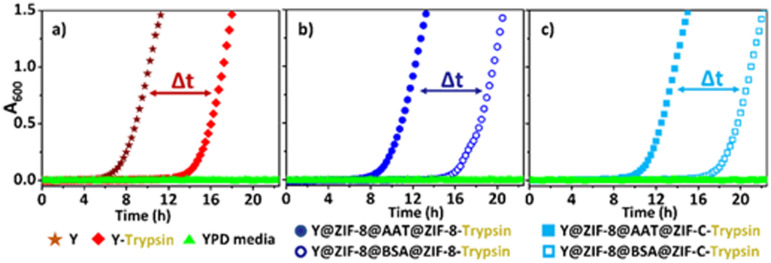
Comparison of cell proliferation (OD600) between: uncoated Y cells exposed and non-exposed to trypsin (a). Y cells released from Y@ZIF-8@AAT@ZIF-8 and Y@ZIF-8@BSA@ZIF-8 and exposed to trypsin during the release process (b). Y cells released from Y@ZIF-8@AAT@ZIF-C and Y@ZIF-8@BSA@ZIF-C and exposed to trypsin during the release process (c).

Overall, we demonstrate that a multilayer approach can be used to coat living cells and immobilize bioactive proteins (antitrypsin, AAT). While affording a homogeneous coating, the multistep process prevents the undesired formation of distinct AAT@ZIF-8 and Y@ZIF-8 particles. By tuning the crystalline phase of the outermost shell (*i.e.* ZIF-8 and ZIF-C), we could select two different release profiles for AAT from Y@ZIF-8@AAT@ZIF-8, Y@ZIF-8@AAT@ZIF-C. Finally, we demonstrated that the fast dissolution of a bio-composite ZIF shell triggers the release of cells while mitigating hostile extracellular conditions.

The authors acknowledge support from the Australian Research Council DP200102411, BioTechMed-Graz Young Researcher Group Program, TU Graz for the Lead Project (LP-03), the European Research Council under the European Union's Horizon 2020 Program (FP/2014–2020)/ERC Grant Agreement (771834—POPCRYSTAL).

## Conflicts of interest

There are no conflicts to declare.

## Supplementary Material

CC-058-D2CC03072A-s001

CC-058-D2CC03072A-s002

CC-058-D2CC03072A-s003
