# Supplementary material for: Multi-layered ZIF-coated cells for the release of bioactive molecules in hostile environments
Source: Chem Commun (Camb). 2022 Aug 1;58(72):10004–7. doi: 10.1039/d2cc03072a (PMC9453912; doi:10.1039/d2cc03072a)
Supplement: CC-058-D2CC03072A-s001 [file CC-058-D2CC03072A-s001.pdf]

Electronic Supplementary Material (ESI) for ChemComm.
This journal is © The Royal Society of Chemistry 2022

Supporting Information

Multi-layered ZIF-coated cells for the release of bioactive molecules in hostile environments

Lei Gan,[a§] Miriam de J. Velásquez-Hernández,[a§] Anita Emmerstorfer-Augustin,[b] Peter Wied,[a] Heimo Wolinski,[c] Simone Dal Zilio,[d] Marcello Solomon,[a] Weibin Liang,[e] Christian Doonan*[e] and Paolo Falcaro*[a]

To whom correspondence should be addressed.

Emails: paolo.falcaro@tugraz.at, christian.doonan@adelaide.edu.au

Author contributions:

PF and CD developed the idea, planned experiments and, with MJV-H, supervised the experimental work. LG, MJV-H, investigated/optimized experimental conditions and performed material characterizations. MS, WL performed initial experiments. PW, AE-A, and HW supported biological experiments and characterized the material with CLSM. SDZ performed morphological investigations with SEM.

Materials

Saccharomyces Cerevisiae (Baker's yeast), yeast extract, zinc acetate dihydrate, 2-methylimidazole (HmIM), D-(+)-glucose, disodium ethylenediaminetetraacetic acid (Na_2EDTA), Bovine Serum Albumin (BSA) (lyophilized powder, >96%), trypsin from porcine pancreas and α1-Antitrypsin (AAT) from human plasma were purchased from Sigma Aldrich and used without further purification. Cell counting was performed using a hemocytometer purchased from Thoma using standard protocols.[1] Samples were mixed using a thermomixer. Cell growth media was prepared by using yeast/peptone media supplemented with 2% glucose and a mixture of adenine, uracil and tryptophan (YPD).[2] Phosphate buffer stock solution (20 mM, pH = 6.5, 500 mL) was prepared by first dissolving $Na_2HPO_4 \cdot 7H_2O$ (957.9 mg, 3.57 mmol) in distilled water (400 mL), then adding $NaH_2PO_4 \cdot 2H_2O$ (886.8 mg, 5.68 mmol). The pH of the solution was adjusted to a final pH of 6.5 using HCl or NaOH. The final volume of the buffer was adjusted to 500 mL.

Synthetic methods

Formation of Y@ZIF-8. This protocol was modified from Liang et al. [3] Dried yeast cells (2 mg) were cultured in 2 mL of YPD, under sterile conditions. The resultant mixture was incubated under stirring (400 rpm) at 30 °C for 18 h. The grown yeast was washed with deionized water (3 × 2 mL) and resuspended in an aqueous solution of HmIM (640 mM, 5 mL), followed by the addition of an aqueous solution of zinc acetate dihydrate (40 mM, 5 mL). The reaction mixture was shaken for 10 min (400 rpm) to yield the formation of Y@ZIF-8. The resultant powdery material was washed with deionized water (DI) (3 × 2 mL), resuspended in DI water (1 mL), and stored at 4 °C until needed.

Formation of Y@ZIF-8@BSA. Three batches of Y@ZIF-8 samples (9 mg) were mixed and resuspended in DI water (0.9 mL). Then, an aqueous solution of BSA (1.5 mg·mL^{-1}, 0.1 mL) was added to the Y@ZIF-8 dispersion. The mixture was shaken for 15 min to yield the formation of Y@ZIF-8@BSA. The resultant material was isolated by centrifugation.

Formation of Y@ZIF-8@BSA@ZIF-8. Y@ZIF-8@BSA was resuspended in DI water (1 mL). Then, 0.33 mL of the Y@ZIF-8@BSA suspension was mixed with an aqueous solution of HmIM (3765 mM, 0.17 mL), followed by the addition of an aqueous solution of zinc acetate dihydrate (40 mM, 0.5 mL). This mixture was shaken for 10 min (400 rpm) to yield the formation of Y@ZIF-8@BSA@ZIF-8. The resultant powdery material was washed with DI water (3 × 2 mL), and recovered by centrifugation.

Formation of Y@ZIF-8@BSA@ZIF-C. The composite Y@ZIF-8@BSA@ZIF-C was obtained following a similar procedure described for Y@ZIF-8@BSA@ZIF-8. First, Y@ZIF-8@BSA was resuspended in DI water (1 mL). Then, 0.33 mL of the Y@ZIF-8@BSA suspension was mixed with a solution of HmIM (941 mM, 0.17 mL), followed by the addition of zinc acetate solution (80 mM, 0.5 mL). This mixture was shaken for 60 min (400 rpm) to yield the formation of Y@ZIF-8@BSA@ZIF-C. Finally, the powdery material was washed with DI water (3 × 2 mL) and recovered by centrifugation.

Formation of Y@ZIF-8@AAT. The formation of Y@ZIF-8@AAT was obtained following the same procedure as described for Y@ZIF-8@BSA, but by using an aqueous solution of AAT (1.5 mg·mL^{-1}, 0.1 mL) instead of BSA.

Formation of Y@ZIF-8@AAT@ZIF-8 and Y@ZIF-8@AAT@ZIF-C. The composites Y@ZIF-8@AAT@ZIF-8 and Y@ZIF-8@AAT@ZIF-C were obtained following the same synthetic procedure as described for Y@ZIF-8@BSA@ZIF-8 and Y@ZIF-8@BSA@ZIF-C; respectively.

Table S1. Different synthetic conditions were explored for the formation of the second MOF coatings. The growth of different ZIF topologies was tuned by varying the concentration of the MOF precursors and the metal: ligand ratio. The conditions used for cell encapsulation were selected based on the formation of pure ZIF phases (blue represents sod-ZIF-8, cyan represents ZIF-C).

Zn^{2+}:HmIM	Zn^{2+} (4 mM)	Zn^{2+} (8 mM)	Zn^{2+} (20 mM)	Zn^{2+} (40 mM)
1:4	ZIF-C (low crystallinity)	ZIF-C (low crystallinity)	ZIF-C (low crystallinity)	ZIF-C
1:8	ZIF-C (low crystallinity)	ZIF-C (low crystallinity)	Mixture phases (80%*dia*+20% ZIF-C)	Mixture phases (90%*dia*+10% ZIF-C)
1:16	Mixture phases (70%*dia*+10% U14+20% ZIF-C)	Mixture phases (80%*dia*+20% ZIF-C)	Mixture phases (90%ZIF-8+10% ZIF-C)	Mixture phases (90%ZIF-8+10% ZIF-C)
1:32	Mixture phases （90%*dia*+10% ZIF-C)	Mixture phases (90%*dia*+10% ZIF-C)	ZIF-8	Mixture phases (90%ZIF-8+10% ZIF-C)

Characterization

Powder X-ray diffraction (PXRD)

PXRD data was collected over the 4–40° 2θ range with a 0.01° step size and a 1°/min scan rate on a Rigaku SmartLab diffractometer using a D/teX Ultra 250 detector (45 kV, 20 mA, 2.5° soller slits) using Cu-Kα (λ = 1.5406 Å) radiation.

UV-Vis spectroscopy

Bradford assay and OD600 measurements were performed on a Thermo ScientificTM NanoDropTM UV-Vis spectrophotometer.

Fourier-transform infrared spectroscopy (FTIR)

FTIR was performed using an alpha Bruker spectrometer using ATR mode (128 scans, 2 cm^{-1} resolution).

Scanning electron microscope-focused ion beam (SEM-FIB)

The SEM-FIB characterization was conducted using a ZEISS LEO 1540XB, equipped with a Ga ion FIB column (V = 30KV). The samples were previously coated with a thin conductive layer of Au-Pd (nominal thickness: 20 nm) deposited by sputter coater (Polaron SC7620). The images were acquired using a High Efficiency In-lens Detector. The cross sectioning has been achieved in low current mode (20-50 pA) to avoid the damage of the cell.

Confocal laser scanning microscopy (CLSM)

CLSM imaging was performed using a Leica SP8 confocal microscope (Leica Microsystems Inc., Germany) with spectral detection and a HC PL APO CS 63x NA 1.2 W CORR objective. FITC was excited at 488 nm and emission detected between 500-550 nm using a noise-less hybrid photon detector. 3D data was acquired with 45 × 45 × 120 nm sampling. Deconvolution was performed using Huygens Professional (Scientific Volume Imaging Inc. The Netherlands); the classic maximum likelihood image restoration method was applied using a theoretical point-spread-function (SNR: 20; 10 iterations). Brightness and contrast of deconvolution data was modified for representation of the images (same values for all images) using the open-source software Fiji. A region of interest of each 18 × 18 μm was cropped from the larger original data sets (45 × 45 μm in size).

Optical microscopy

Optical microscope images were collected on a ZEISS AxioScope A1 using 20× objective lenses.

Release tests

The cumulative release of the encapsulated protein (BSA and AAT) was assessed by UV-Vis spectroscopy. In this work, we studied two different media to trigger the release of the target protein at room temperature. The fast release was observed by exposing the cell-composites to a chelating agent EDTA (0.1 M, 1 mL). The controlled release was observed using a phosphate buffer solution (20 mM, 1 mL, pH = 6.5).

The cumulative release was recorded by suspending the MOF bio-composites (4 mg) either in EDTA or phosphate buffer. The samples were shaken using an orbital mixer. At regular intervals, the mixture was vortexed for 3 s and centrifuged for 30 s. Then, an aliquot of the supernatant (0.5 mL) was taken and replaced with the same volume of fresh EDTA or phosphate buffer. The supernatant aliquot removed from the reaction was mixed with Bradford reagent (1 mL) and analyzed by UV-Vis spectroscopy at 595 nm. All the experiments were performed in triplicates.

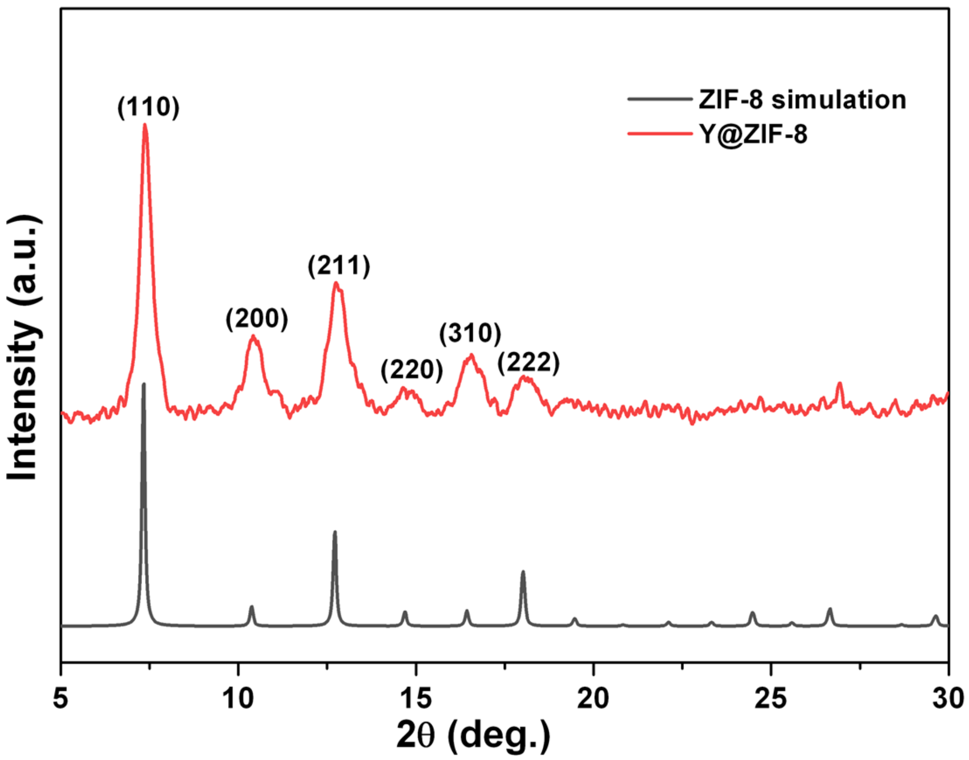

Fig. S1 X-ray pattern collected from **Y@ZIF-8** composite.

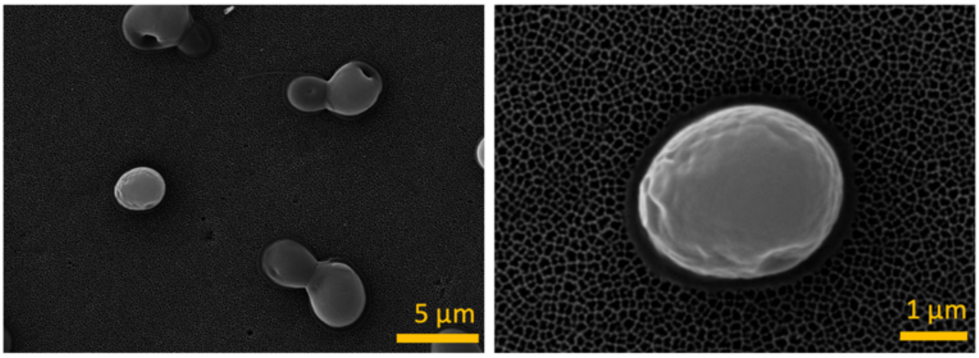

Fig. S2 SEM images of uncoated Yeast cells (**Y**)

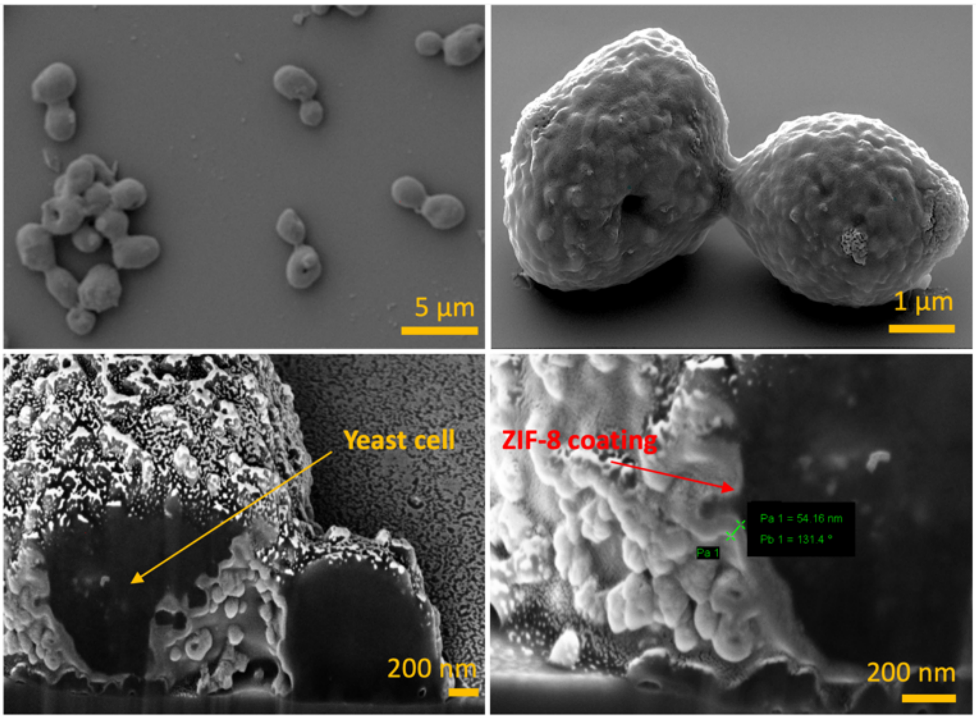

Fig. S3 SEM images of **Y@ZIF-8** (up). Cross-section of **Y@ZIF-8** obtained by gallium focused ion beam (Ga-FIB) (down left). The average thickness of fist ZIF-8 coating is 60 ± 20 nm (down-right). Notes: 1) the film thickness is the result from preparation conditions that lead to an enhanced protection of yeast cells from trypsin, 2) the low accuracy from the SEM estimation of the thickness is reflected in the error of the measurement.

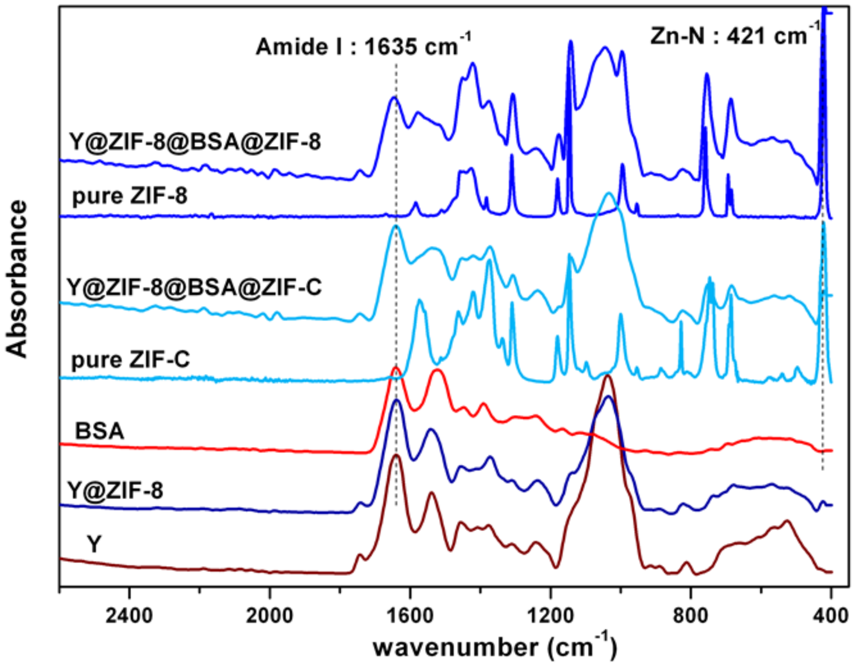

Fig. S4 Comparison between IR spectra of **Y@ZIF-8@BSA@ZIF** and **Y@ZIF-8** composites and the controls (Y = yeast, ZIF= ZIF-8, ZIF-C)

Adsorption efficiency optimisation using BSA as model protein

The optimisation study was carried out by mixing a stock solution of BSA ($S0_{BSA}$ = 1.5 mg mL^{-1}, 100 µL) with different amounts of Y@ZIF-8 resuspended in DI water (900 µL). Thus, the resultant concentration of BSA was kept constant (0.15 mg mL^{-1}), while the amount of Y@ZIF-8 composite varied systematically by adding incremental volumes of Y@ZIF-8 stock suspension ($S0_{Y@ZIF-8}$ =10 mg mL^{-1}) (see Table S2 for details).

The resultant mixture was shaken for 15 min at room temperature. Then, the solids were collected by centrifugation, and the remaining amount of BSA in the supernatant was determined by Bradford assay. Finally, the adsorption efficiency was calculated with the following formula.

$$AE\% = \frac{([BSA]_i - [BSA]_f) * 100}{[BSA]_i}$$

Where:

$[BSA]_i$ = Initial concentration of BSA (0.15 mg mL^{-1})

$[BSA]_f$ = Concentration of BSA in the supernatant after the adsorption process

Table S2 summarises the total amount of BSA adsorbed by varying the Y@ZIF-8 composite soaked into the protein solution. The collected data suggest that the maximum amount of BSA that can be adsorbed by 1 mg of Y@ZIF-8 composite is *ca.* 17 µg of protein (See Fig. S5).

Table S2. Experimental conditions used for the adsorption efficiency optimization

	Premix volume = 900 µL				
Sample	$S0_{Y@ZIF-8}$ (µL)	H_2O (µL)	$S0_{BSA}$ (µL)	[BSA] in the supernatant (mg mL^{-1})	% AE
S1	150	750	100	0.125	16.66
S2	300	600	100	0.1	33.33
S3	600	300	100	0.05	66.66
S4	900	0	100	0	100

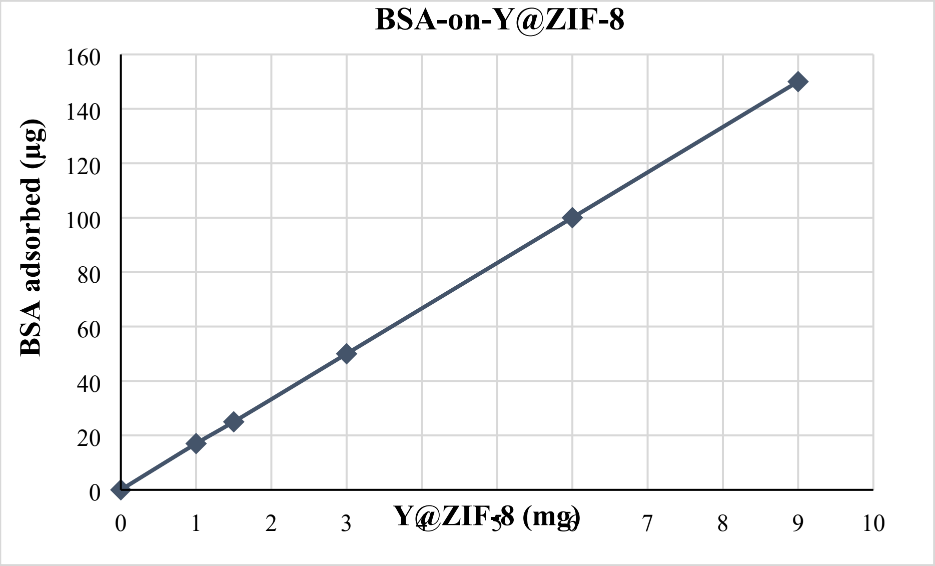

Fig. S5 Linear correlation between the mass of BSA adsorbed and the mas of Y@ZIF-8 soaked into the protein solution.

From the optimization with BSA, we selected 1 mg of Y@ZIF-8 composite to adsorb *ca.* 17 µg of AAT.
Based on both UV-vis adsorption of the protein (280 nm) and Bradford assay from the supernatant (vide supra, e.g. S1, S2) we could confirm the 100% AAT adsorption.

Next, to examine any possibility of protein detachment from Y@ZIF-8 during the growth of the second MOF shell, we performed a micro-Bradford assay on the reaction media collected after the Y@ZIF-8@protein@ZIF synthesis (protein = BSA, AAT; ZIF=ZIF-8, ZIF-C). The calibration curve for BSA was first made using six standard solutions containing BSA or AAT at varying concentrations (0, 0.01, 0.02, 0.03, 0.04, 0.05 mg mL^{-1}) (Fig. S6 and Fig. S15). An aliquot of each sample (0.5 mL) was mixed with Bradford reagent (1.0 mL). The resultant mixture was left for 5 min at room temperature, followed by UV-vis analysis at 595 nm (Table S3 and Table S5). A similar procedure was used to assess the amount of BSA or AAT released into the reaction medium during the second coating process (Fig. S16). All experiments were performed in triplicates. The collected data confirms that neither BSA nor AAT is released from the MOF surfaces to the reaction medium during the second coating synthesis.

Table S3 Micro-Bradford assay of BSA standard solutions and protein detachment assessment

	BSA Concentration mg mL^{-1}	Absorbance			
		@595 nm			Average
BSA in water for calibration					
Sample 1	0	0	0	0	0.00
Sample 2	0.01	0.13	0.13	0.13	0.13
Sample 3	0.02	0.29	0.29	0.29	0.29
Sample 4	0.03	0.42	0.42	0.42	0.42
Sample 5	0.04	0.51	0.51	0.51	0.51
Sample 6	0.05	0.63	0.63	0.63	0.63
**Supernatant** collected from Y@ZIF-8@BSA@ZIF-8 reaction medium	0	0	0	0	0
**Supernatant** collected from Y@ZIF-8@BSA@ZIF-C reaction medium	0	0	0	0	0

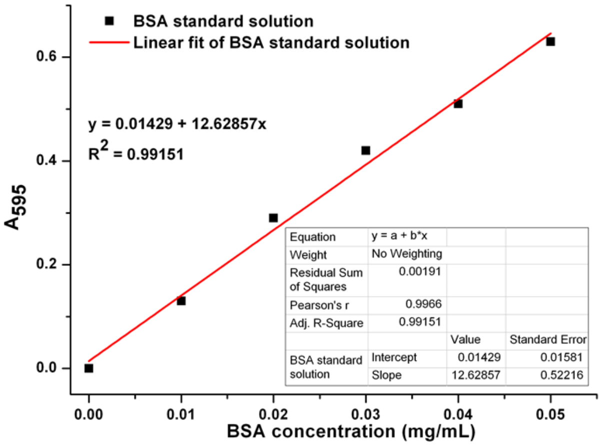

Equation	y = a + b*x		
Weight	No Weighting		
Residual Sum of Squares	0.00191		
Pearson's r	0.9966		
Adj. R-Square	0.99151		
		Value	Standard Error
BSA standard solution	Intercept	0.01429	0.01581
	Slope	12.62857	0.52216

Fig. S6 Calibration curve of obtained from the Bradford assay of BSA standard solutions (λ_{max} = 595 nm)

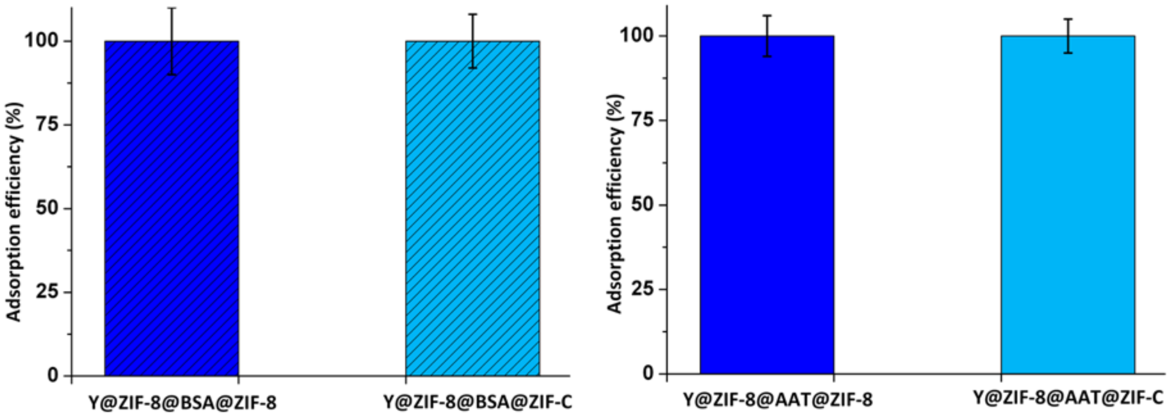

Fig. S7 Adsorption efficiency (AE) of BSA in **Y@ZIF-8@BSA@ZIF** (ZIF = ZIF-8, ZIF-C) (left) and AAT in **Y@ZIF-8@AAT@ZIF** (ZIF = ZIF-8, ZIF-C) (right).

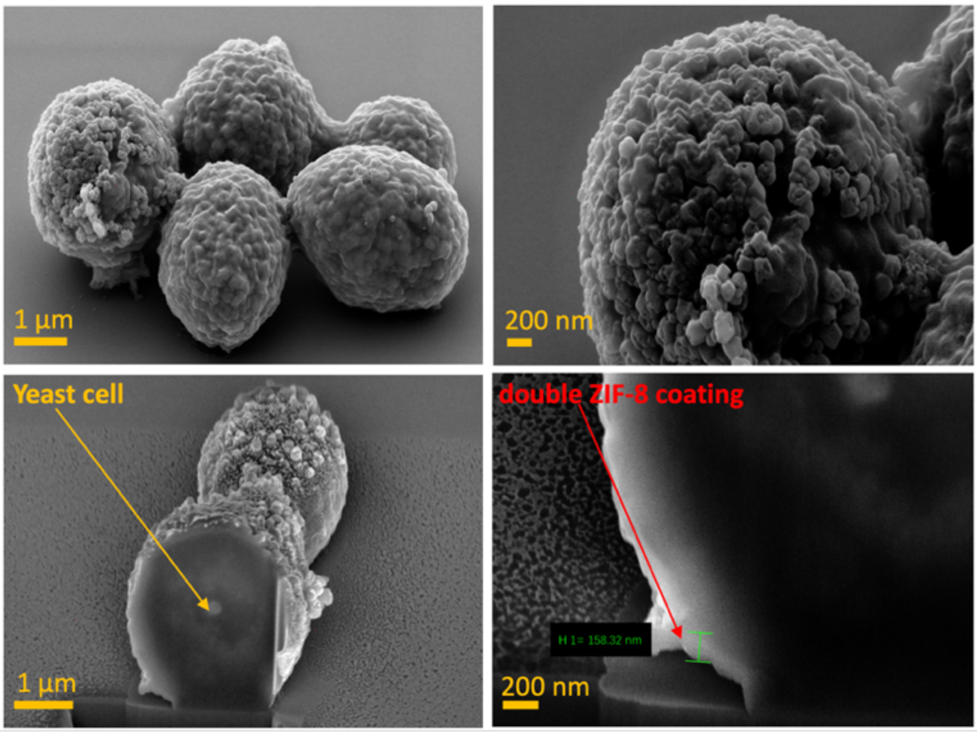

Fig. S8 SEM images of **Y@ZIF-8@BSA@ ZIF-8** (up). Cross-section of **Y@ZIF-8@BSA@ ZIF-8** obtained by gallium focused ion beam (Ga-FIB) (down-left). The average thickness of ZIF-8 double coating is 102 ± 20 nm (down-right).

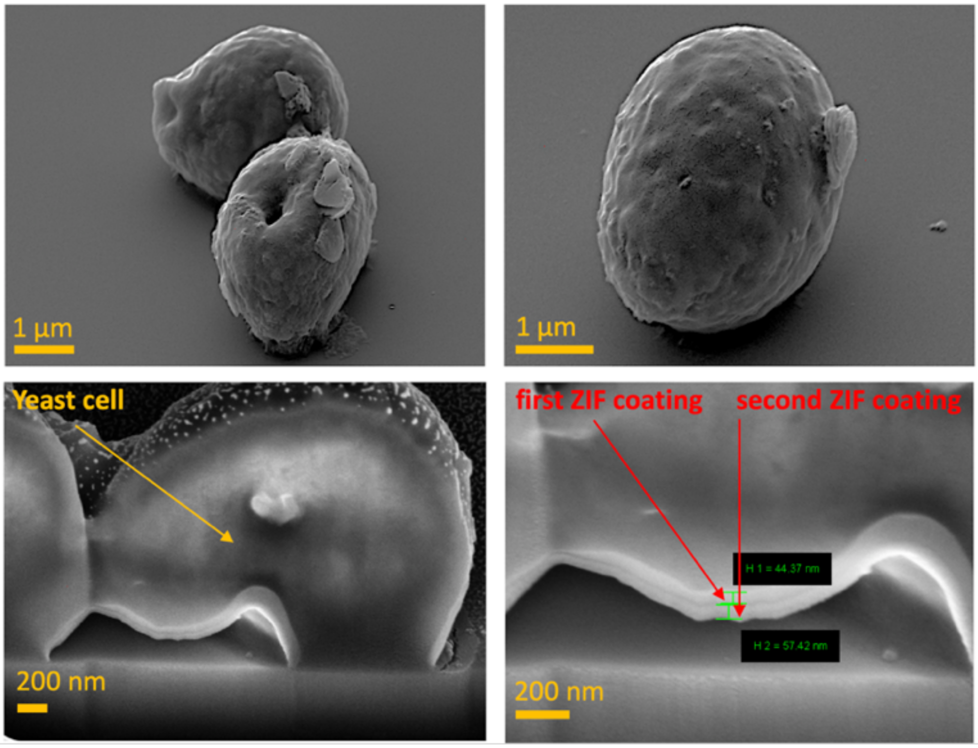

Fig. S9 SEM images of **Y@ZIF-8@BSA@ ZIF-C** (up). Cross-section of **Y@ZIF-8@BSA@ ZIF-C** obtained by gallium focused ion beam (Ga-FIB) (down-left). The average thickness of ZIF double coating is 95 ± 10 nm (down-right).

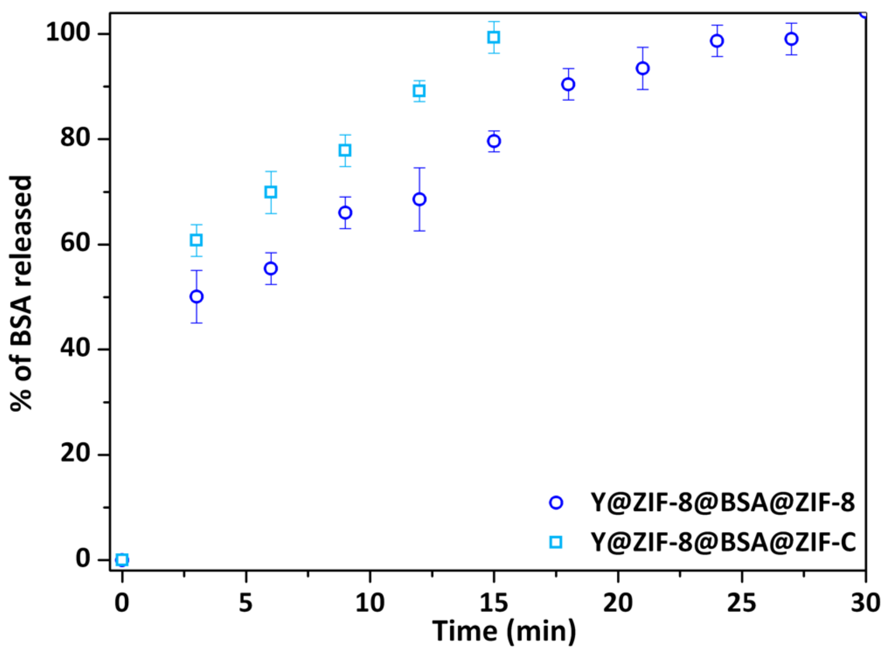

Fig. S10 Release profiles of BSA from **Y@ZIF-8@BSA@ZIF** (ZIF = ZIF-8; ZIF-C) composites obtained by soaking 4 mg of the material in 1 mL of EDTA solution.

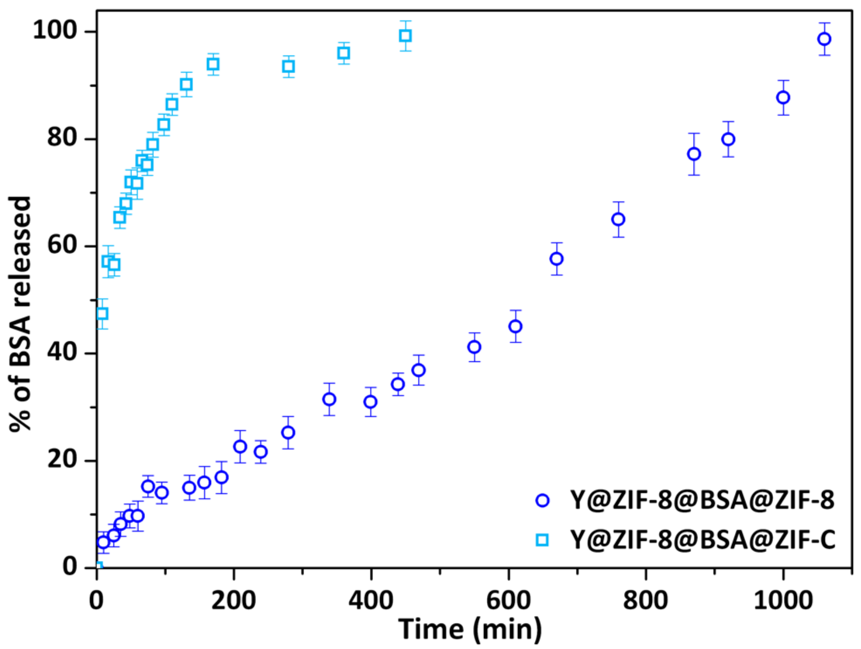

Fig. S11 Release profiles of BSA from **Y@ZIF-8@BSA@ZIF** (ZIF = ZIF-8; ZIF-C) composites obtained by soaking 4 mg of the material in 1 mL of phosphate buffer solution (20 mM, pH 6.5, 25 ^{o}C).

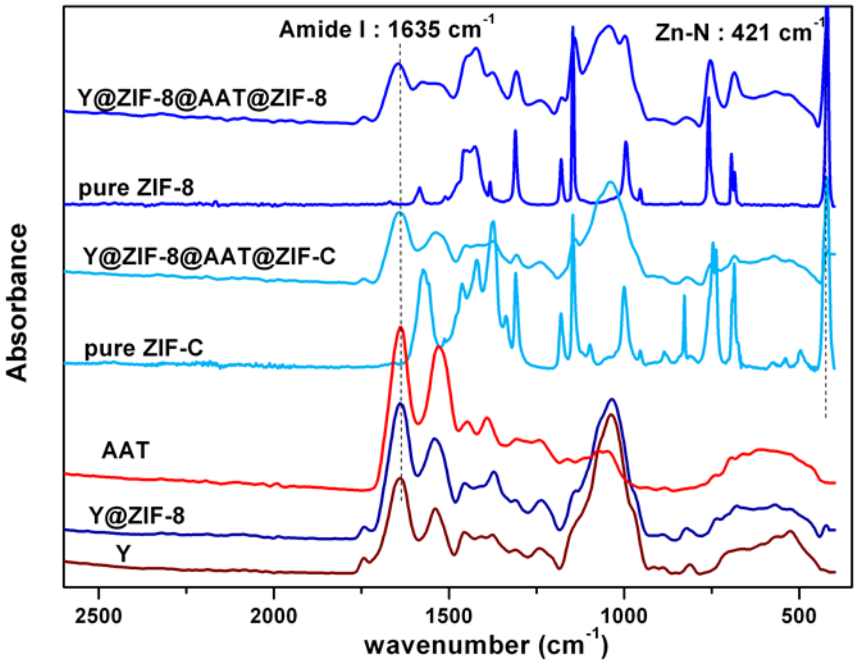

Fig. S12 Comparison between IR spectra of **Y@ZIF-8@AAT@ZIF** and **Y@ZIF-8** composites and the controls (Y = yeast, ZIF = ZIF-8, ZIF-C)

Assessment of AAT adsorption onto Y@ZIF-8 composites

- **Experiment 1: Direct AAT quantification using UV-vis spectroscopy**

  The quantification of proteins in aqueous solutions can be determined by measuring the UV-vis absorbance at 280 nm. The protein absorbance at 280 nm is primarily due to the presence of amino acids with aromatic residues, for example, tryptophan and tyrosine.[4-6] This method is fast and straightforward because it does not require additional reagents. The protein absorption at 280 nm was employed to evaluate the amount of Alpha-1-antitrypsin (AAT) adsorbed onto yeast@ZIF-8:

  a) firstly, the Y@ZIF-8 composite was resuspended in an aqueous solution of AAT (0.15 mg mL^{-1}). The resultant mixture was shaken for 15 min;
  b) then, the solid material was separated by centrifugation;
  c) finally, the supernatant was collected and the concentration of the remaining protein was determined by UV-Vis spectroscopy (λ_{max} = 280 nm).

Fig. S13 (see also below) shows the UV-vis spectra obtained from the AAT (0.15 mg mL^{-1}) solution before and after the protein adsorption process. The collected data indicate the absence of AAT in the supernatant and thus the adsorption of AAT onto the Y@ZIF-8 biocomposite.

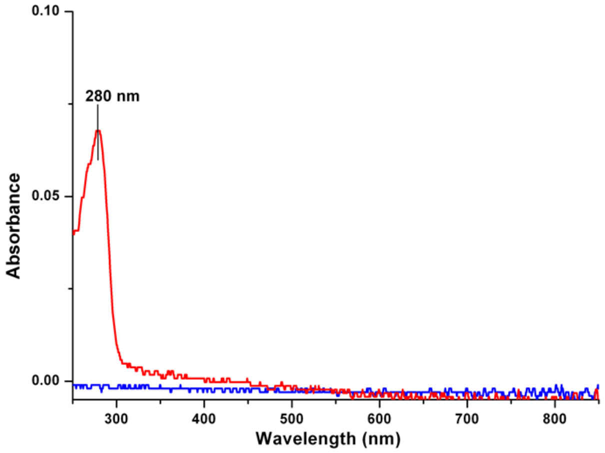

Fig. S13 Direct UV-vis measurements of the AAT solution (0. 15 mg/mL) before (red) and after (blue) the AAT adsorption onto Y@ZIF-8 composite.

- **Experiment 2: AAT quantification by Bradford assay.**

The Bradford assay is typically used to determine the concentration of proteins in complex mixtures, such as cell extracts.[7, 8] The protein (i.e. AAT) determination was performed following the Sigma-Aldrich standard protocol.[9] Briefly, a calibration curve for AAT was made by preparing solutions with different AAT concentrations (0, 0.06, 0.08, 0.10, 0.12, 0.15 mg·mL^{-1}). An aliquot (50 µL) of each sample was mixed with Bradford reagent (1.5 mL). The resultant mixture was left for 5 min at room temperature prior to analysis by UV-Vis spectroscopy λ_{max} = 595 nm (Table S4 and Fig. S14). A similar procedure was used to determine the amount of AAT in the supernatant of Y@ZIF-8 after the adsorption process. The collected data indicate the absence AAT in solution. This confirms the quantitative adsorption of AAT onto the Y@ZIF-8 biocomposite with a standard protein assay.

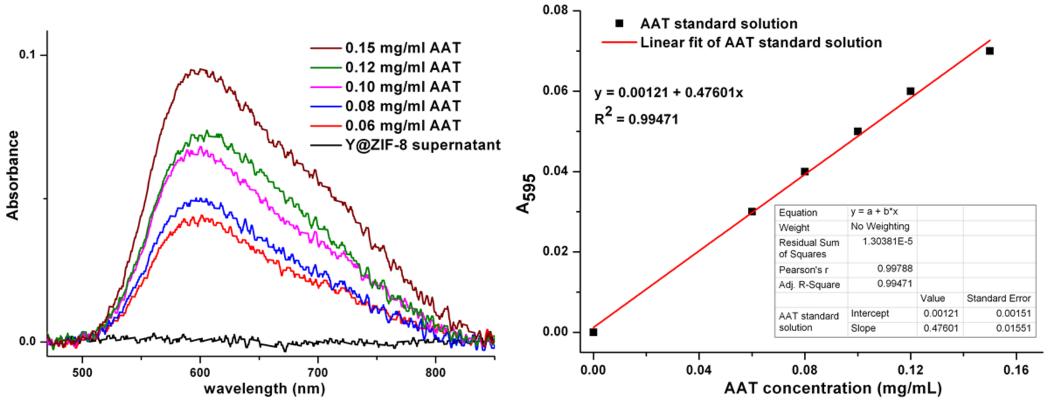

Fig. S14 UV-vis spectra obtained from the AAT standard solutions and supernatant measured in Pierce Coomassie Plus (Bradford) reagent (left). Calibration curve obtained from the Bradford assay of AAT standard solutions (λ_{max} = 595 nm) (right).

Table S4 Bradford assay of AAT samples: UV-vis absorbance obtained from the AAT standard solutions and the supernatant collected from AAT-on-Y@ZIF-8 reaction medium (λ_{max} = 595 nm)

	Concentration of AAT (mg mL^{-1})	Absorbance			
		@595 nm			Average
AAT in water for calibration					
Sample 1	0	0	0	0	0.00
Sample 2	0.06	0.03	0.03	0.03	0.03
Sample 3	0.08	0.04	0.04	0.04	0.04
Sample 4	0.1	0.05	0.05	0.05	0.05
Sample 5	0.12	0.06	0.06	0.06	0.06
Sample 6	0.15	0.07	0.07	0.07	0.07
Supernatant of AAT-on-Y@ZIF-8	0	-0.01	0	0	0

Table S5 Micro-Bradford assay of AAT standard solutions and protein detachment assessment

	AAT Concentration mg mL^{-1}	Absorbance			
		595 nm			Average
AAT in water for calibration					
S1	0	0	0	0	0.00
S2	0.01	0.14	0.14	0.14	0.14
S3	0.02	0.25	0.25	0.25	0.25
S4	0.03	0.33	0.33	0.33	0.33
S5	0.04	0.48	0.48	0.48	0.48
S6	0.05	0.59	0.59	0.59	0.59
**Supernatant** collected from Y@ZIF-8@AAT@ZIF-8 reaction medium	0	0	0	0	0
**Supernatant** collected from Y@ZIF-8@AAT@ZIF-C reaction medium	0	0	0	0	0

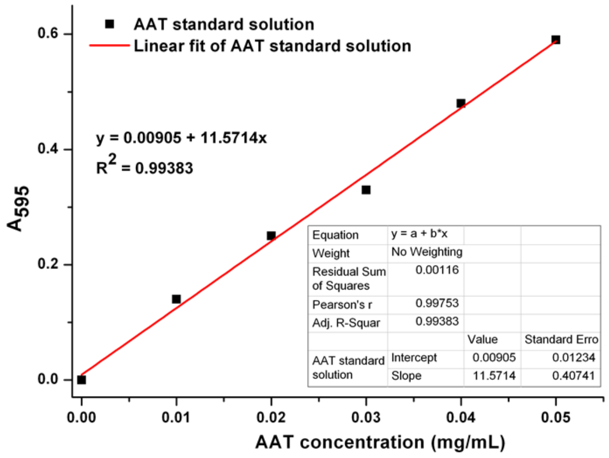

Fig. S15 Calibration curve of obtained from the Bradford assay of AAT standard solutions (λ_{max} = 595 nm)

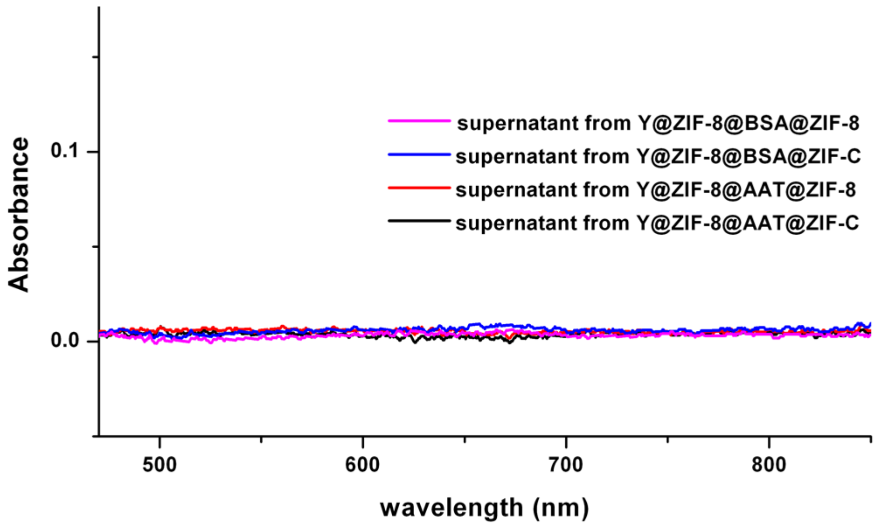

Fig. S16 The UV-Vis spectra obtained from Y@ZIF-8@protein@ZIF reaction media (protein= BSA, AAT; ZIF=ZIF-8, ZIF-C)

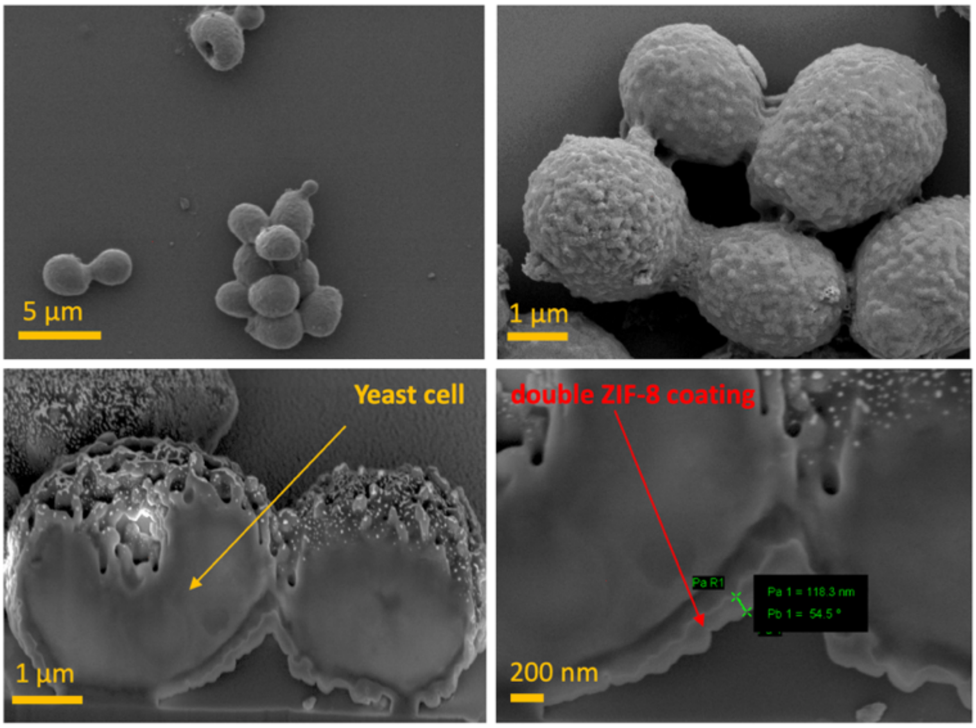

Fig. S17 SEM images of **Y@ZIF-8@AAT@ ZIF-8** (up). Cross-section of **Y@ZIF-8@AAT@ ZIF-8** obtained by gallium focused ion beam (Ga-FIB) (down-left). The average thickness of double ZIF-8 coating is 270 ± 20 nm (down-right).

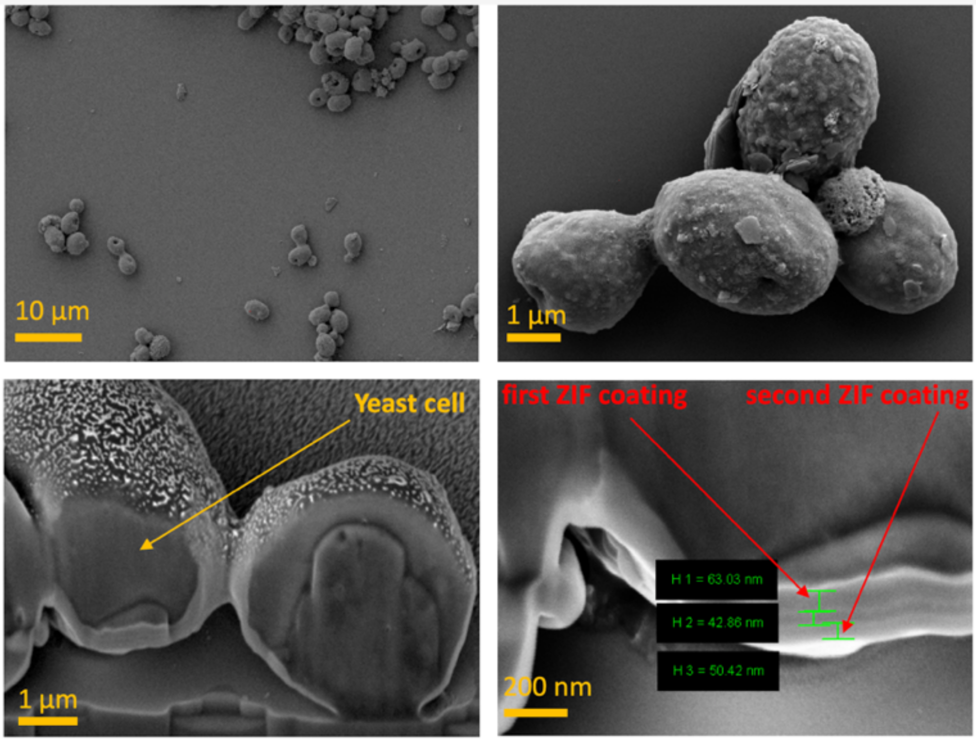

Fig. S18 SEM images of **Y@ZIF-8@AAT@ ZIF-C** (up). Cross-section of **Y@ZIF-8@AAT@ ZIF-C** obtained by gallium focused ion beam (Ga-FIB) (down-left). The average thickness of ZIF double coating is 125 ± 40 nm (down-right).

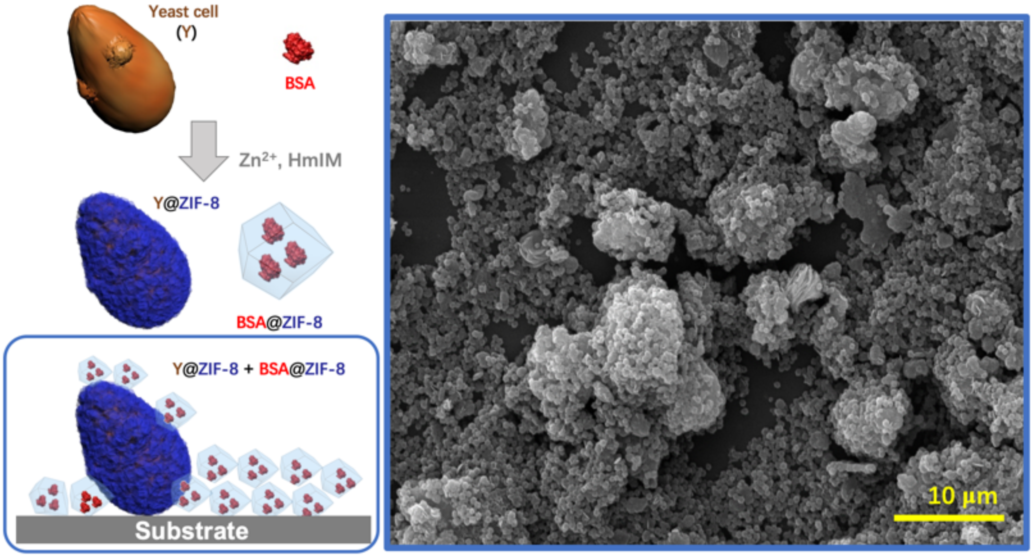

Fig. S19 Schematic representation and SEM of the product obtained from the single-step encapsulation of Yeast and BSA within ZIF-8. This strategy results in a mixture of Y@ZIF-8 and BSA@ZIF-8 particles separated; this is clearly visible when depositing the sample on the substrate, and investigate the morphology by SEM.

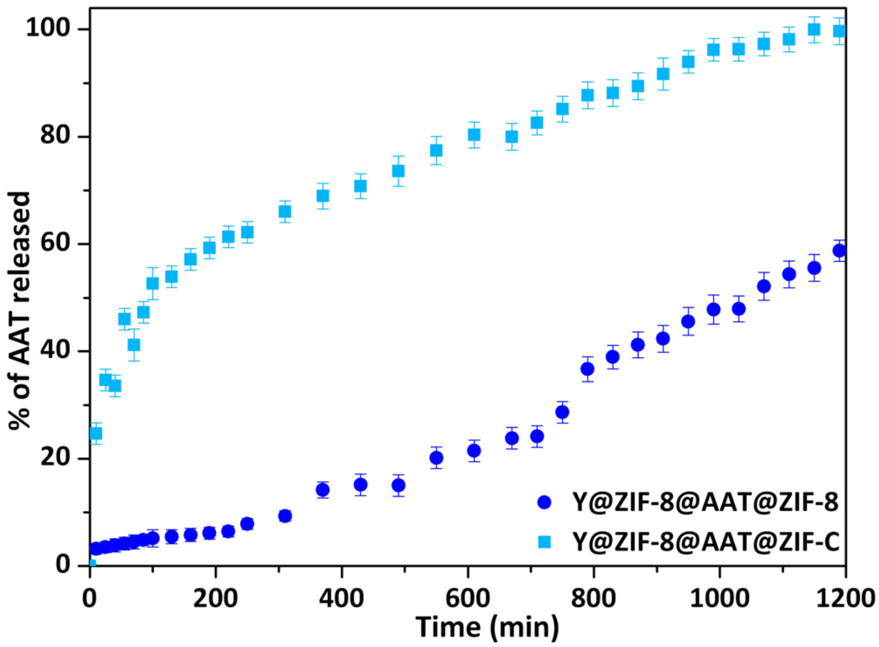

Fig. S20 Release profiles of AAT from **Y@ZIF-8@AAT@ZIF** (ZIF = ZIF-8; ZIF-C) composites obtained by soaking 4 mg of the material in 1 mL of phosphate buffer solution (20 mM, pH 6.5, 25 °C).

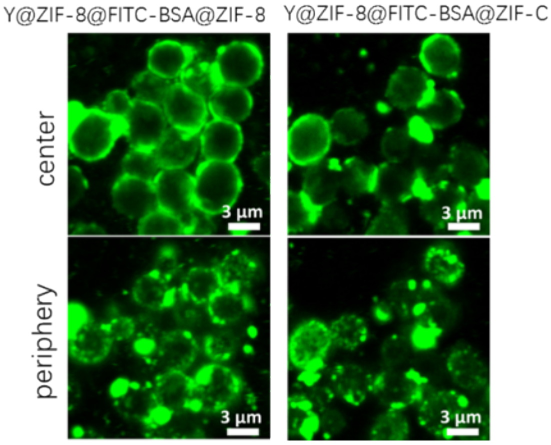

Fig. S21 Non-deconvoled single confocal optical sections taken from the center of **Y@ZIF-8@FITC-BSA@ZIF** (ZIF = ZIF-8, ZIF-C) composites (up) and from the periphery of **Y@ZIF-8@FITC-BSA@ZIF** (ZIF = ZIF-8, ZIF-C) composites (down).

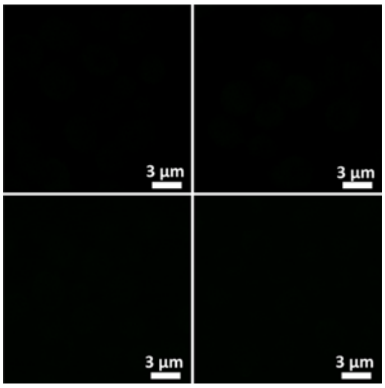

Fig. S22 The control samples prepared with pure BSA and pure AAT are shown here. Single confocal optical sections of **Y@ZIF-8@BSA@ZIF-8** (top-left), **Y@ZIF-8@BSA@ZIF-C** (top-right), **Y@ZIF-8@AAT@ZIF-8** (down-left) and **Y@ZIF-8@AAT@ZIF-C** (down-right).

Protease inhibitor activity of AAT released from Y@ZIF-8@AAT@ZIF (ZIF=ZIF-8, ZIF-C)

The protease inhibitor activity of AAT could be determined by exposing a solution of AAT to trypsin. Then, the enzymatic activity of trypsin was determined by using a colorimetric assay and Nα-Benzoyl-L-arginine ethyl ester (BAEE) as a substrate.[9] Briefly, the Y@ZIF-8@AAT@ZIF (ZIF = ZIF-8, ZIF-C) sample was suspended in EDTA (0.1 M, 1 mL). The resultant mixture was shaken for 1 h (400 rpm). Afterward, the sample was centrifuged, and the supernatant was separated from the uncoated cells. Trypsin (0.25 mg·mL^{-1}, 25 μL) was then added to the supernatant and the mixture stirred on a thermomixer (800 rpm) for 30 min at 4 °C. This solution was added to a quartz cuvette containing BAEE (6.88 mg·mL^{-1}) dissolved in sodium phosphate monobasic buffer (67 mM, pH 7.6, 25 °C, 25 μL) and HCl (1 mM, 17 μL). The sample was mixed by inversion and measured continuously by UV-Vis spectroscopy at 253 nm over 400 seconds. Both the activity of trypsin not exposed to AAT, and the activity of trypsin (0.25 mg·mL^{-1}, 25 μL) exposed to a solution of lyophilized powder of AAT (0.05 mg·mL^{-1}, 1 mL) were examined as experimental controls.

Slow Release of AAT from Y@ZIF-8@AAT@ZIF (ZIF=ZIF-8, ZIF-C).

To test the gradual inhibition of trypsin caused by the slow release of AAT, five samples of Y@ZIF-8@AAT@ZIF samples were dispersed, separately, in phosphate buffer solution (20 mM, 1 mL, pH = 6.5) and stirred in a thermomixer (400 rpm). Each sample was centrifuged at different time points 2, 4, 6, 8, and 10 h. The supernatant

(1 mL) was added to trypsin (0.25 mg·mL^{-1}, 25 μL); the resultant mixture was stirred for further 30 min at 4 °C. The enzymatic activity of trypsin was determined by the trypsin colorimetric assay (*vide supra*).

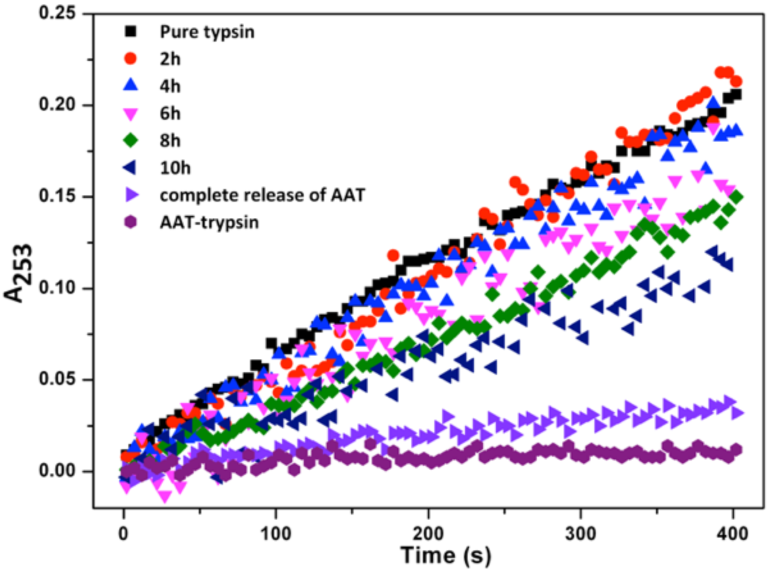

Fig. S23 Enzymatic activity of trypsin exposed to AAT fully released from **Y@ZIF-8@AAT@ZIF-8.** This plot also demonstrates how the slow release of AAT leads to the gradual inhibition of trypsin activity.

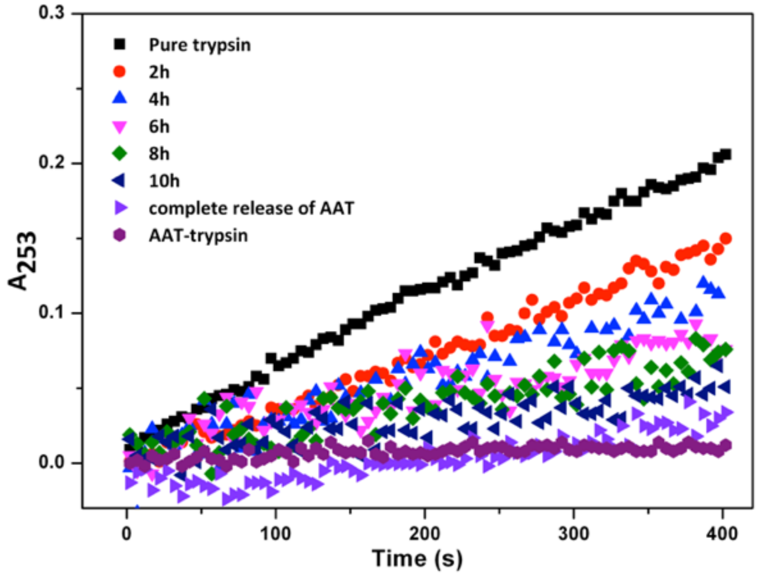

Fig. S24 Enzymatic activity of trypsin exposed to AAT fully released from **Y@ZIF-8@AAT@ZIF-C.** This plot also demonstrates how the slow release of AAT leads to the gradual inhibition of trypsin activity.

Cell proliferation experiment

The composite samples were suspended in an aqueous solution containing EDTA (0.1 M, **1 mL**) and trypsin (0.25 mg·mL^{-1}, 25 μL). The mixture was shaken for 4 h, centrifuged and washed with DI water (3 × 2 mL). The released cells were dispersed in H_2O (20 mL) and then an aliquot (2 µL) was taken and added to the growth media YPD (2 mL). The cells' growth at room temperature was monitored by Optical Density measurements at 600 nm (OD600). As a control, the cell growth of naked cells exposed and non-exposed to trypsin was monitored by OD600. Briefly, non-encapsulated yeast cells were exposed to EDTA (0.1 M, **1 mL**) with trypsin (0.25 mg·mL^{-1}, 25 μL) or without trypsin for 4 h. The mixture was shaken for 4 h, centrifuged and washed with DI water (3 × 2 mL). The cells were

then resuspended in H_2O (20 mL) and then an aliquot (2 µL) was taken and added to growth media YPD (2 mL). The cell growth at room temperature was monitored by OD600.

References

1. D. Cadena-Herrera, J. E. Esparza-De Lara, N. D. Ramírez-Ibañez, C. A. López-Morales, N. O. Pérez, L. F. Flores-Ortiz and E. Medina-Rivero, *Biotechnol. Rep.*, 2015, **7**, 9-16.
2. C.-W. Hung, J. Y. Martínez-Márquez, F. T. Javed and M. C. Duncan, *Sci. Rep.*, 2018, **8**, 1-16.
3. K. Liang, J. J. Richardson, J. Cui, F. Caruso, C. J. Doonan and P. Falcaro, *Adv. Mater.*, 2016, **28**, 7910-7914.
4. H. Edelhoch, *Biochemistry*, 1967, **6**, 1948-1954.
5. S. C. Gill and P. H. Von Hippel, *Anal. Biochem.*, 1989, **182**, 319-326.
6. C. N. Pace, F. Vajdos, L. Fee, G. Grimsley and T. Gray, *Protein Sci.*, 1995, **4**, 2411-2423.
7. N. J. Kruger, *The protein protocols handbook*, 2009, 17-24.
8. F. He, *Bio-protocol*, 2011, e45-e45.
9. https://www.sigmaaldrich.com/technical-documents/protocols/biology/enzymatic-assay-oftrypsin.html.
